# Supplementary material for: Complexome profiling of the Chlamydomonas psb28 mutant reveals TEF5 as an early PSII assembly factor
Source: Plant Cell. 2025 Mar 17;37(6):koaf055. doi: 10.1093/plcell/koaf055 (PMC12164590; doi:10.1093/plcell/koaf055)
Supplement: koaf055_Supplementary_Data [file koaf055_supplementary_data.zip › psb28_interactive.zip!/plots/intensity/qualityPlots/peptides/NUOP5_Cre08.g378050.t1.2.html]

### Cre08.g378050.t1.2

Trivial name: NUOP5  
SeqCoverage [median]: 76.90  
Mass: 15.27 kDa  
  
 

| Sequence | MissedCleavage | Mass [Da] | wtIntensity | muIntensity | Score |
| --- | --- | --- | --- | --- | --- |
| DPSELQAAATSVEAR | 0 | 1543.75 | 3084.84 | 5710.29 | 131.69 |
| ERTMYDDYAANAEEPIIYKR | 2 | 2447.14 | 1331.47 | 827.00 | 74.95 |
| EVDPTGQAK | 0 | 943.46 | 25673.52 | 20678.51 | 176.75 |
| NHTYMYNLK | 0 | 1182.55 | 8040.43 | 3301.76 | 100.25 |
| NNDDSASYKEWQDFLDVAEKDR | 2 | 2644.17 | 1487.59 | 0.00 | 34.66 |
| RDPSELQAAATSVEAR | 1 | 1699.85 | 30132.59 | 24573.88 | 157.07 |
| SGELGGLSPLA | 0 | 999.52 | 3923.12 | 0.00 | 124.08 |
| SYTRRDPSELQAAATSVEAR | 2 | 2207.09 | 883.21 | 1416.77 | 39.39 |
| TAGFVWR | 0 | 835.43 | 3939.28 | 8005.91 | 126.24 |
| TMYDDYAANAEEPIIYK | 0 | 2005.90 | 464.80 | 0.00 | 58.67 |
| TMYDDYAANAEEPIIYKR | 1 | 2162.00 | 11959.72 | 15808.54 | 130.77 |
| VKSGELGGLSPLA | 1 | 1226.69 | 2937.62 | 12114.49 | 84.17 |
|  |  |  | 93858.2 | 92437.1 |  |
